# Cultural Adaptation and Validation of the Portuguese Version of the CANHELP Lite Bereavement Questionnaire

**DOI:** 10.3390/healthcare8010027

**Published:** 2020-02-02

**Authors:** Alexandra Pereira, Amélia Ferreira, Ana Rita Abrantes, Cristiana Gomes, Joana Saraiva, Laetitia Teixeira, Daren K. Heyland, José Martins, Sara Pinto, Olga Fernandes

**Affiliations:** 1 Abel Salazar Biomedical Institute, Community Care Unit of Lousada, 4620-848 Lousada, Portugal; amelia.leite.ferreira@gmail.com; 2 End of Life Project, 3046-841 Coimbra, Portugal; rita.dig@hotmail.com; 3 Clínica Vila Ramadas, 2460-355 Leiria, Portugal; cristigomes1@gmail.com; 4 Centro Hospitalar Universitário de Coimbra, 3000-075 Coimbra, Portugal; joana.s.saraiva88@gmail.com; 5 Abel Salazar Biomedical Institute, R. Jorge de Viterbo Ferreira 228, 4050-313 Porto, Portugal; lcteixeira@icbas.up.pt; 6 Department of Critical Care Medicine, Queen’s University, Kingston, Ontario ON K7L 3N, Canada; Dkh2@queensu.ca; 7 Medical-Surgical Nursing Department, Nursing School of Coimbra, 3046-841 Coimbra, Portugal; amelia.ferreira123@sapo.pt; 8 Escola Superior de Saúde de Santa Maria, Center for Health Technology and Services Research (CINTESIS), NursID, 4049-024 Porto, Portugal; sara.o.pinto@gmail.com; 9 Escola Superior de Enfermagem do Porto, Center for Health Technology and Services Research (CINTESIS), NursID, 4200-072 Porto, Portugal; olgafernandes@esenf.pt

**Keywords:** end-of-life care, satisfaction, validation study, outcome measure

## Abstract

Background: Satisfaction with care is an important outcome measure in end-of-life care. Validated instruments are necessary to evaluate and disseminate interventions that improve satisfaction with care at the end of life, contributing to improving the quality of care offered at the end of life to the Portuguese population. The purpose of this study was to perform a cross-cultural adaptation and psychometric analysis of the Portuguese version of the CANHELP Lite Bereavement Questionnaire. Methods: Methodological research with an analytical approach that includes translation, semantic, and cultural adaptation. Results: The Portuguese version comprised 24 items. A panel of experts and bereaved family members found it acceptable and that it had face and content validity. A total of 269 caregivers across several care settings in the northern region of Portugal were recruited for further testing. The internal consistency analysis of the adapted instrument resulted in a global alpha value of 0.950. The correlation between the adapted CANHELP questionnaire and a global rating of satisfaction was of 0.886 (*p* < 0.001). Conclusions: The instrument has good psychometric properties. It was reliable and valid in assessing caregivers’ satisfaction with end-of-life care and can be used in both clinical and research settings.

## 1. Introduction

The quality of care provided to individuals at the end of life, and to their family members, has become an important health and social issue across the world [1]. Quality end-of-life care is considered a right for all citizens and the responsibility of every government [2]. Thus, improving care at the end of life is an international priority [3,4,5] and it is key to improving the quality of life for patients and their families [2].

Understanding how care is perceived by family members across care settings is essential to identify specific domains of care that need to be improved [1] and that influence how end-of-life care is delivered and experienced [6].

Although satisfaction with care has its limitations as an endpoint and does not equate to the overall quality of care, it is considered an important domain within the overall quality of end-of-life care [6].

The comprehension of families’ levels of satisfaction with end-of-life care is important to improve individual care and to introduce quality improvement initiatives to the health system. The use of these instruments will also allow families, policymakers, and the public to hold individuals and organizations accountable for the quality of care provided to patients at the end of life [6,7,8]. 

Care provided at the end of life is receiving growing attention across the world, particularly in Portugal. Although Portugal is considered to have a generalized provision of palliative care [9], it has one of the highest rates of adults in need of palliative care [10] and there are still inequities in the distribution of, and access to, palliative care [11].

Nevertheless, knowledge about the care provided at the end of life in Portugal across care settings is still limited [12]. Thus, there is also little evidence regarding the quality of care at the end of life. To evaluate the quality of care at the end of life, it is necessary to use standardized and validated measurement tools, including those that measure families’ satisfaction as an important indicator of the quality of care [6,13,14]. 

Recent research has been conducted aiming to identify basic quality indicators for palliative care services [8,15,16]. This evidence shows that family satisfaction regarding different aspects of palliative care is considered to be an important outcome measure in three different palliative care quality domains, namely the structure and process of care, psychological and psychiatric aspects of care, and care of the imminently dying patient [8].

A recent bibliometric study regarding the postgraduate academic publications in Portugal in the field of palliative care over the past few years showed that few methodological studies were conducted in Portugal [17]. Methodological studies are important for the development of adequate measurement instruments to assess palliative care-related quality indicators [18]. Evidence shows that the lack of reliable instruments is a factor hindering the assessment of quality indicators in palliative care [19,20].

Currently, there are some tools available worldwide to specifically assess families’ satisfaction of end-of-life care [21]. However, in Portugal, there is only one validated tool that specifically measures families’ satisfaction with end-of-life care: FAMCARE [22]. A few studies using small samples have been conducted in Portugal to examine the effectiveness of the FAMCARE tool [23,24]. 

Although FAMCARE was initially developed to measure family satisfaction with advanced cancer care [25], it is often used in palliative care research. However, it has considerable limitations as it was designed to measure the satisfaction levels of family members who received home-based palliative care, it does not always include all the aspects of the care provided, and the psychometric properties have not yet been sufficiently well-established because of the small sample size in the validation testing [26]. Also, the use of FAMCARE with bereaved family members is not well-established. Although FAMCARE has been used in bereaved family members in international studies [27], this has not been done or tested in Portugal. For the reasons, it was necessary to validate an instrument that could be used across care settings and applied to bereaved family members.

The CANHELP (Canadian Health Care Evaluation Project) instruments were developed to measure satisfaction with end-of-life care [6,28]. CANHELP is a self-reporting instrument that was developed and validated for patients at the end of life and their family members to assess satisfaction with a variety of actionable items. The CANHELP Bereavement Questionnaire is a 43-item instrument with two items about overall satisfaction with care and 41 items that fall into one of six quality of care subscales: doctor and nurse characteristics; illness management; health service characteristics; communication and decision-making; you and your relationships with others; and spirituality and meaning. Although the instrument showed good internal consistency reliability, the length of the interview may limit its uptake and clinical utility. 

A shorter version of the instrument was developed [29,30]. The CANHELP Lite Bereavement Questionnaire is a 24-item instrument with two items about overall satisfaction with care and 22 items that fall into one of five quality of care subscales: relationship with the doctors; characteristics of doctors and nurses; illness management; communication and decision making; and your involvement. Responses to each item are scored on a five-point scale ranging from ‘not at all satisfied’ (1) to ‘completely satisfied’ (5) [31]. 

The aim of this study was to perform the cultural adaptation and validation of the Portuguese version of the CANHELP Lite Bereavement Questionnaire.

## 2. Materials and Methods 

### 2.1. Study Design

This was a methodological study including translation, semantic, and cultural adaptation, and the evaluation of the psychometric properties, according to the guidelines proposed by Beaton et al. [32].

### 2.2. Translation, Cross-Cultural Adaptation and Validation Processes

The process of cultural adaptation looks at both language (translation) and cultural adaptation issues, while preparing a questionnaire for use in another setting. The following stages composed the translation and semantic and cultural adaptation of the instrument [32]:

1. Linguistic translation of the instrument into European Portuguese by three translators, native in Portugal and fluent in American English. Two translators were unaware of the concept under study, but the third was an expert in the palliative care field.
2. Synthesis and review of the first translation.
3. Back-translation of the reviewed version by two other translators, natives of the USA and fluent in Portuguese. They did the semantic analysis and had an agreement result of 100.0%.
4. Review of all translations by a panel of experts (one expert in research and methodological studies, two experts in palliative care, and one expert in bereavement, in addition to all translators and back-translators). The original developers of the questionnaire were in close contact with the panel of experts during this part of the process.
5. Assessment of face and content validity through a pre-test on a sample of 20 bereaved family members, which aimed to verify the understanding of the items, evaluate the ease of using the response set, and estimate the response time. The instrument was easily understood, and the bereaved family members answered when requested. The average answer time was 10 min for the entire questionnaire. After the face validity of the instrument was analyzed, its psychometric properties were examined. The literature proposes the inclusion of 5–10 participants per item for the sample [33]. The inclusion of 10 participants per item was estimated and the final sample was composed of 269 bereaved family members.

### 2.3. Participants, Setting and Procedure

The participants were bereaved family members knowledgeable about the healthcare provided during the last three months of life to an adult decedent (aged 18 years and older). To identify potential participants, the death certificates of all adults (aged 18 years and older) that were available in the national electronic death certificate registration were analyzed. Records of decedents where the cause of death codes were associated with external or accidental causes, suicides, pregnancy complications, or medical and surgical complications, as well as death certificates that had an uncertain cause of death were excluded.

In Portugal, death certificates do not have information regarding family members and thus, it was necessary to cross information and to search for a phone number on the health system national registration using the health national registration number provided on the death certificate. A first phone call was made to inform the participants about the study’s aims and the voluntary nature of their participation. They were also given guarantees of data confidentiality and anonymity. Informed consent was obtained from each bereaved family member. After that, a phone interview was scheduled at a date of the bereaved family member’s preference. Subsequently, the instrument was applied through a phone interview conducted by trained interviewers. All interviews were conducted three to 12 months after death. As the period of bereavement is recognized as a sensitive time, this interval was decided to respect the family bereavement period but also to guarantee that the memory of the experience was recent enough to be considered reliable.

### 2.4. Data Analysis

Descriptive analysis was performed through means and standard deviation, or numbers and percentages of the demographic and clinical variables. The Kolmogorov–Smirnov test was performed to check the distribution of the variables enrolled. A bivariate correlation by Pearson was used.

The assumptions used in the original version were followed and the internal consistency of the scale was obtained using Cronbach’s alpha. Cronbach’s alpha if item deleted and corrected item-total correlation were also obtained. According to the literature, alpha values under 0.50 are unacceptable, from 0.50 to 0.60 are questionable, from 0.60 to 0.70 are acceptable, from 0.70 to 0.80 are good, from 0.80 to 0.90 are very good, and over 0.90 are considered excellent [34,35].

Criterion-related validity was determined through the concurrent use of a single-item, global rating of caregivers’ general satisfaction with the care received in the last three months of life (scored between 1 and 5, meaning that 1 was the worst level of satisfaction possible and 5 the best level of satisfaction possible). This option is justified by the fact that there isn’t a validated tool in Portugal to assess bereaved family members’ satisfaction with the care received in the last months of life regardless of the existing pathology or setting of care. This was performed using the Pearson correlation coefficient.

Convergent–discriminant validity was performed using an exploratory factorial analysis considering the principal components method with orthogonal rotation (varimax method with Kaiser normalization).

It was not possible to perform test–retest reliability, as the purpose of the instrument was to assess the bereaved families’ satisfaction with the care received in the last three months of life. As this might be considered a sensitive matter, we believe that respecting human dignity should have priority over the interests of the research.

The data were analyzed using SPSS, version 23, for Windows. A level of significance of 0.05 was assumed.

### 2.5. Ethical Procedures

First, a research protocol was designed. It was then submitted, analyzed, and approved by the Portuguese Data Protection Authority (number 9607/2016), ethics committee (number P361/09-2016), and the assistant secretary of state and health. A collaboration protocol was also signed between the research team and the Directorate General of Health.

## 3. Results

### 3.1. Study Population

All 269 participants were bereaved Portuguese family members from the north region recruited through the identification provided in the health system national registration after analysis of the death certificates. The majority of the participants were female (78.4%) and married (59.5%), and were most often daughters of the deceased (47.6%). The average age was 58.2 years (sd = 13.3 years; minimum = 18 years and maximum = 91 years). The interview was conducted, on average, 295.93 days after the death of the relative (sd = 76.6 days; minimum = 93 days and maximum = 364 days). 

### 3.2. Reliability

Considering the 24 items of the scale, the analysis of internal consistency reliability revealed an alpha of 0.950. The global alpha when removing items were also analyzed (Table 1). Although Cronbach’s alpha would increase if items 14, 16, and 23 were deleted, it was decided to retain them, as the alpha obtained is already considered excellent.

The English and Portuguese item description of the CANHELP Lite Bereavement Questionnaire is available in Table 2.

### 3.3. Construct Validity

During the translation and cultural adaptation phase, it was observed that the scale was answered quickly and that there were no suggestions for alterations in items or terms considered inadequate or unnecessary, which shows a good understanding and acceptance of the items by the participants.

Concurrent validity was determined using the Pearson correlation coefficient, and a value of 0.886 (*p* < 0.001) was obtained.

Regarding convergent–discriminant validity, an exploratory factorial analysis considering the principal components method with orthogonal rotation (varimax method with Kaiser normalization) was performed. The initial exploratory factor analysis of our study proposed four factors (Table 3). 

**Figure 1:**
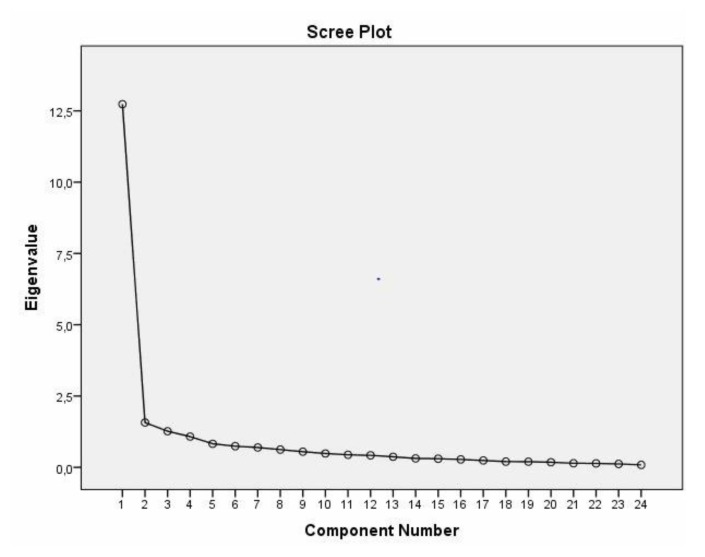
Scree plot extracted from the exploratory factor analysis of the Portuguese version of the CANHELP Lite Bereavement Questionnaire.

The CANHELP Lite Bereavement Questionnaire proposes the evaluation of five quality subscales, namely relationship with the doctors, characteristics of doctors and nurses, illness management, communication and decision making, and your involvement. Despite these considerations, the instrument remains unidimensional as the first factor explains 53.061% of the total variance of the items and the second factor explains 6.545%, indicating unidimensional adequacy (Figure 1).

## 4. Discussion

The current study provides evidence for the reliability and validity of the Portuguese version of the CANHELP Lite Bereavement Questionnaire. To the best of our knowledge, this study is the first cross-cultural adaptation and psychometric analysis of this instrument in Portugal. A rigorous methodology for the translation, cross-cultural adaptation, and validation processes following the recommended guidelines has been used in this study.

The cross-cultural adaptation and psychometric testing of this instrument were achieved satisfactorily following the recommendations of the international guidelines [36,37]. Regarding this, the Portuguese version of the CANHELP Lite Bereavement Questionnaire showed satisfactory psychometric properties. The face and content validity of the instrument was acceptable, which indicates that all items are relevant for the construct measurement. The internal consistency analysis was satisfactory (Cronbach’s alpha = 0.950), suggesting excellent homogeneity of the items. These values are in line with those reported in the original studies [29,30].

In terms of concurrent criterion-related validity, there was adequate correlation with the single-item global rating of caregiver satisfaction with the care received in the last three months of life, which might indicate that the Portuguese version of the CANHELP Lite Bereavement Questionnaire is adequate to measure satisfaction with the care received in the last months of life.

An after-death assessment of the caregivers’ satisfaction with the care received in the last months of life would allow us to identify, evaluate, and disseminate interventions that improve satisfaction with care at the end of life, which would also, indirectly, improve the quality of care at the end of life. For that, it is necessary to have valid and reliable instruments. Thus, we believe that the translation and validation of the Portuguese version of the CANHELP Lite Bereavement Questionnaire will contribute to improving care at the end of life in the Portuguese population.

This study followed a rigorous methodology and was developed across care settings, which allow us to be confident regarding the generalizability of the results produced here. Nevertheless, there are some limitations. Effects related to memory might be considered a limitation of this study, as it is a retrospective evaluation by caregivers. In this context, the evaluation of satisfaction with care could be affected by the amount of time between the death and the evaluation. Proxy reports after end-of-life care can be obtained at varying time intervals post-death. Regarding this, it was decided to conduct the interview three to 12 months after the patient’s death. This is a conventional time frame for evaluating this construct, as similar periods of time were used in other studies [38,39,40]. However, we recognize that other intervals may provide more accurate data. Future research should explore the optimal timing for the collection of data from bereaved caregivers. Also, the test–retest reliability was not performed, which is the reason why the instruments’ sensitivity to change was not evaluated.

## 5. Conclusions

The Portuguese version of the CANHELP Lite Bereavement Questionnaire is a valid and reliable instrument for the assessment of satisfaction with end-of-life care and can be used in both clinical and research settings. 

This is the first instrument available in Portugal to assess bereaved family members’ satisfaction with end-of-life care and it could be very important as it might be a valuable contribution to improve the Portuguese health system.

## Figures and Tables

**Table 1:** Descriptive statistics and internal consistency for the items.

Item	Mean	Standard Deviation	Corrected Item-Total Correlation	Cronbach’s Alpha if Item is Deleted
1	3.70	0.982	0.843	0.946
2	3.52	1.049	0.847	0.945
3	3.53	1.020	0.811	0.946
4	3.01	1.044	0.837	0.946
5	3.72	0.891	0.841	0.946
6	3.64	0.973	0.813	0.946
7	2.85	0.966	0.764	0.947
8	3.96	0.915	0.589	0.949
9	3.58	0.841	0.668	0.948
10	3.17	1.010	0.751	0.947
11	3.72	0.877	0.732	0.947
12	3.28	0.903	0.713	0.947
13	3.55	0.947	0.794	0.946
14	1.85	0.760	0.277	0.951
15	3.58	0.933	0.619	0.948
16	2.68	1.146	0.416	0.951
17	3.60	0.873	0.773	0.947
18	3.30	0.920	0.722	0.947
19	3.00	0.977	0.756	0.947
20	2.69	1.105	0.645	0.948
21	2.06	0.910	0.646	0.948
22	3.80	1.181	0.573	0.949
23	2.99	1.673	0.106	0.960
24	3.17	0.912	0.637	0.948

**Table 2:** Item description of the CANHELP Lite Bereavement Questionnaire

Item	English Description | Portuguese Description
1	In general, how satisfied are you with the quality of care your relative received? | Em geral, qual o seu nível de satisfação com a qualidade dos cuidados recebidos pelo seu familiar?
2	In general, how satisfied are you with the way you were treated by the doctors and nurses looking after your relative? | Em geral, qual o seu nível de satisfação com a forma como foi tratado pelos médicos e enfermeiros que cuidaram do seu familiar?
3	How satisfied are you that the doctor(s) took a personal interest in your relative? | Qual o seu nível de satisfação com o interesse pessoal demonstrado pelos médicos perante o seu familiar?
4	How satisfied are you that the doctor(s) were available when you or your relative needed them (by phone or in person)? | Qual o seu nível de satisfação com a disponibilidade demonstrada pelos médicos para si e para o seu familiar quando necessitaram (presencial ou telefonicamente)?
5	How satisfied are you with the level of trust and confidence you had in the doctor(s) who looked after your relative? | Qual o seu nível de satisfação com o nível de confiança que tinha no(s) médico(s) que cuidou(aram) do seu familiar?
6	How satisfied are you that the doctors, nurses, and other health care professionals who looked after your relative were compassionate and supportive of him or her? | Qual o seu nível de satisfação com a compaixão e apoio recebidos pelo seu familiar por parte dos médicos e enfermeiros que dele cuidaram?
7	How satisfied are you that the doctors, nurses, and other health care professionals who looked after your relative were compassionate and supportive of you? | Qual o seu nível de satisfação com a compaixão e apoio recebidos por si por parte dos médicos e enfermeiros que cuidaram do seu familiar?
8	How satisfied are you with the tests that were done and the treatments that were given for your relative’s medical problems? | Qual o seu nível de satisfação com os exames e os tratamentos realizados ao seu familiar?
9	How satisfied are you that physical symptoms (for example: pain, shortness of breath, nausea) your relative had were adequately assessed and controlled? | Qual o seu nível de satisfação com a avaliação e o controlo dos problemas físicos do seu familiar?
10	How satisfied are you that emotional problems (for example: depression, anxiety) your relative had were adequately controlled? | Qual o seu nível de satisfação com o controlo dos problemas emocionais (por exemplo: depressão, ansiedade) do seu familiar?
11	How satisfied are you with the help your relative received with personal care (for example: bathing, toileting, dressing, eating)? | Qual o seu nível de satisfação com os cuidados pessoais (por exemplo: dar banho, ir ao wc, comer) recebidos pelo seu familiar?
12	How satisfied are you that your relative received good care when you were not able to be with him/ her? | Qual o seu nível de satisfação com os cuidados recebidos pelo seu familiar na sua ausência?
13	How satisfied are you that health care workers worked together as a team to look after your relative? | Qual o seu nível de satisfação com a equipa de profissionais de saúde que cuidou do seu familiar?
14	How satisfied are you that you were able to manage the financial costs associated with your relative’s illness? | Qual o seu nível de satisfação com a sua capacidade para gerir os custos económicos associados à doença do seu familiar?
15	How satisfied are you with the environment or the surroundings where your relative was cared for? | Qual o seu nível de satisfação com o ambiente onde o seu familiar foi cuidado?
16	How satisfied are you that the care and treatment your relative received was consistent with his or her wishes? | Qual o seu nível de satisfação com os cuidados e tratamentos recebidos pelo seu familiar, tendo em conta os seus desejos?
17	How satisfied are you that the doctor(s) explained things to you relating to your relative’s illness in an honest manner? | Qual o seu nível de satisfação com a honestidade dos médicos a explicar a situação de doença do seu familiar?
18	How satisfied are you that you received consistent information about your relative’s condition from all the doctors and nurses looking after him or her? | Qual o seu nível de satisfação com a congruência dos médicos a explicar a situação de doença do seu familiar?
19	How satisfied are you that the doctor(s) listened to what you had to say? | Qual o seu nível de satisfação com a forma como os médicos a escutaram?
20	How satisfied are you with discussions with the doctor(s) about where your relative would be cared for (in hospital, at home, or elsewhere) or when her/his condition got worse? | Qual o seu nível de satisfação com as conversas que teve com os médicos sobre o local onde o seu familiar seria cuidado (no hospital, em casa, ou outro local) quando piorasse?
21	How satisfied are you with discussions with the doctor(s) about the use of life sustaining technologies (for example: CPR or cardiopulmonary resuscitation, breathing machines, dialysis)? | Qual o seu nível de satisfação com as conversas que teve com os médicos sobre o uso de técnicas de suporte de vida (por exemplo: reanimação, ventilação assistida, diálise)?
22	How satisfied are you with your role in decision-making regarding your relative’s medical care? | Qual o seu nível de satisfação com o papel que desempenhou nas tomadas de decisão em relação aos cuidados de saúde do seu familiar?
23	How satisfied are you with discussions with your relative about wishes for future care in the event he or she was unable to make those decisions? | Qual o seu nível de satisfação com as conversas que teve com o seu familiar sobre as preferências dele em relação aos cuidados de saúde quando já não estivesse capaz?
24	How satisfied are you that you came to understand what was expected at the end stage of your relative’s illness (for example: in terms of symptoms and comfort measures)? | Qual o seu nível de satisfação com a sua compreensão sobre o que era esperado no estadio final da doença do seu familiar (por exemplo: em termos de sintomas e medidas de conforto)?

**Table 3:** Exploratory factorial analysis considering the principal components method with orthogonal rotation (varimax method with Kaiser normalization).

Item	Component			
	1	2	3	4
1	0.809	0.396		
3	0.771	0.392		
5	0.758	0.456		
13	0.754	0.424		
6	0.744	0.448		
10	0.739		0.363	
9	0.717		0.408	
11	0.715		0.302	
12	0.698			
8	0.690			
2	0.681	0.566		
4	0.679	0.539		
7	0.592	0.534		
15	0.559		0.509	
18	0.358	0.768		
17	0.458	0.741		
19	0.397	0.725		
24	0.357	0.492	0.336	
22	0.397	0.458		−0.456
16			0.738	
20		0.557	0.618	
21		0.541	0.587	
14			0.319	0.677
23			0.405	−0.528
